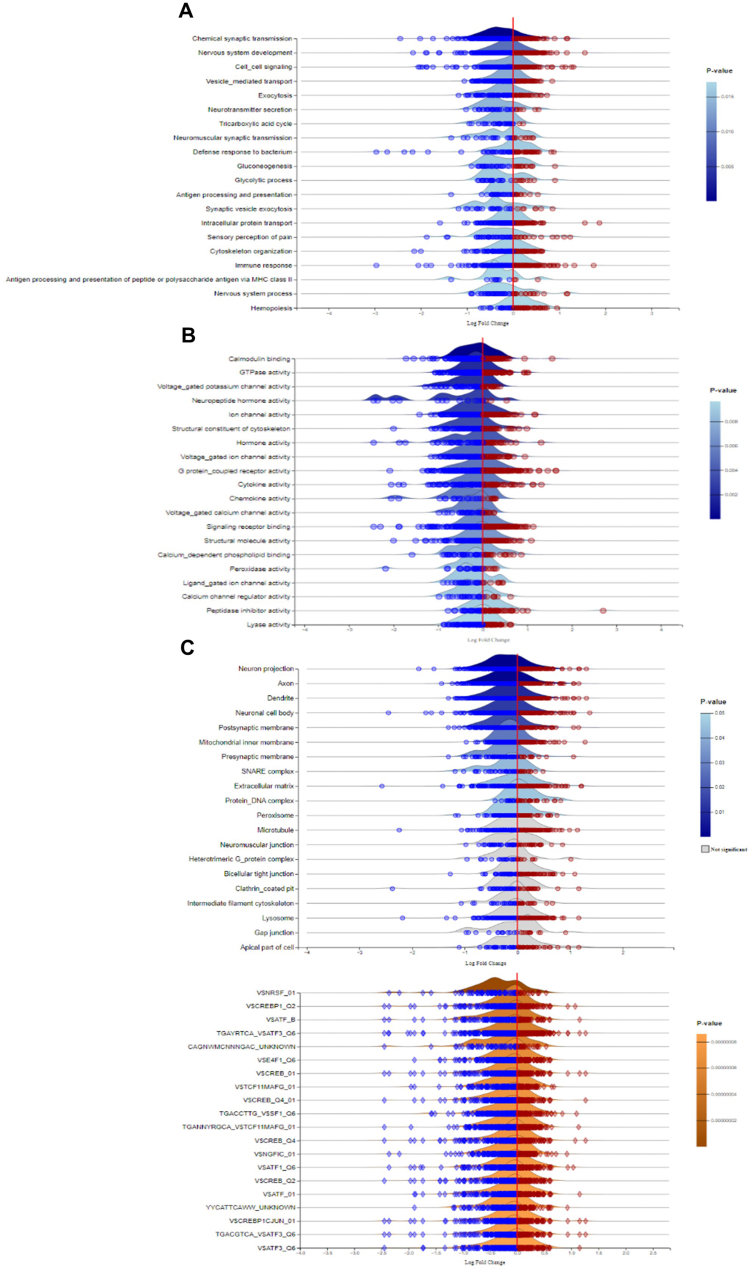# Untangling the complex mechanisms associated with Alzheimer's disease in elderly patients using high-throughput RNA sequencing data and next-generation knowledge discovery methods: Focus on potential gene signatures and drugs for dementia

**DOI:** 10.1016/j.heliyon.2024.e41266

**Published:** 2024-12-18

**Authors:** Hind A. Alkhatabi, Peter Natesan Pushparaj

**Affiliations:** a Department of Biological Science, College of Science, University of Jeddah, Jeddah, Saudi Arabia; b Institute of Genomic Medicine Sciences, Faculty of Applied Medical Sciences, King Abdulaziz University, Jeddah, 21589, Saudi Arabia

**Keywords:** Alzheimer's disease, Dementia, GREIN, ExpressAnalyst, RNA sequencing, Gene set enrichment analysis, Next-generation knowledge discovery, Drugs, Natural products

## Abstract

**Objectives:**

Alzheimer's disease (AD) is a complex neurodegenerative disorder that primarily affects elderly individuals. This study aimed to elucidate the intricate mechanisms underlying AD in elderly patients compared with healthy aged individuals using high-throughput RNA sequencing (RNA-seq) data and next-generation knowledge discovery methods (NGKD), with a focus on identifying potential therapeutic agents.

**Methods:**

High-throughput RNA-seq data were obtained from the Gene Expression Omnibus (GEO) database (accession number: GSE104704). These data were derived from healthy and diseased human brains (eight young healthy brains [young], 10 aged healthy brains [Old], and 12 aged diseased brains [AD]). We used NGKD tools such as GEO RNA-seq Experiments Interactive Navigator (GREIN) to obtain differentially expressed genes (DEGs) by comparing the AD versus Old RNA-seq data and further filtered and normalized to obtain differentially regulated Kyoto Encyclopedia of Genes and Genomes (KEGG), Reactome and Panther pathways using ExpressAnalyst tool. Besides, WebGestalt was used to identify differentially regulated Gene Ontologies (GO) and the pre-ranked Gene Set Enrichment Analysis (GSEA) was performed using GSEA software. The X2K web tool was used to infer upstream regulator networks and X2K Appyter tool for obtaining transcription factors (TFs) and kinase network information. LFW1000 and L1000CDS^2^ tools were used to identify specific drugs that reverse AD-associated gene signatures in elderly patients.

**Results:**

Our study revealed significant downregulation of pathways related to neuroactive receptor-ligand interaction, synaptic vesicle cycle, and neuronal system in elderly individuals with AD. GO analysis showed negative enrichment of functions related to cognition, potassium ion transport, receptor-ligand activity, SNARE binding, and primary lysosomes. The transcription factors SUZ12 and REST, along with increased MAPK signaling, were identified as key regulators of downregulated genes. Several drugs and natural products, including dihydroergocristine, mepacrine, gedunin, amlodipine, and disulfiram have been identified as potential therapeutic agents for reversing AD-associated gene signatures.

**Conclusions:**

This comprehensive analysis of AD in elderly individuals using RNA-seq data and NGKD tools revealed multiple differentially regulated pathways, gene signatures, and potential drugs for dementia treatment. These findings highlight the complex molecular mechanisms underlying AD and provide insights into potential therapeutic strategies. Further research is needed to validate these findings and to develop personalized treatment approaches for AD in elderly patients.

## 1 Introduction

Alzheimer's disease (AD) is a complex and devastating neurodegenerative disorder characterized by progressive cognitive decline, memory loss, and behavioral alterations [1]. It represents the primary etiology of dementia and poses a significant challenge to the global healthcare system [1]. The impact of AD extends far beyond the affected individuals, profoundly affecting families, caregivers, and society. The prevalence of AD is substantial and rapidly increasing, and alarming statistics highlight the urgent need to address this growing health crisis. In the United States alone, an estimated 6.7 million Americans aged ≥65 years are currently affected by AD dementia in the United States alone [1]. This figure is projected to escalate dramatically to 13.8 million by 2060 in the absence of medical advancements to prevent, decelerate, or cure the disease. This situation is of equal concern globally, with over 55 million individuals living with dementia in 2020. Projections indicate nearly doubling of this number every two decades, reaching 78 million by 2030 and 139 million by 2050. AD accounts for an estimated 60–80 % of dementia cases, with the majority of individuals experiencing brain changes attributable to multiple etiologies of dementia [[1], [2], [3]]. Advanced age remains the most significant risk factor for AD, underscoring the complex relationship between ageing and neurodegenerative processes [1].

Aging populations, genetic predispositions, and lifestyle factors such as diabetes, cardiovascular disease, and obesity contribute to the high prevalence of AD [1,4,5]. Age is the most significant risk factor for AD, and as individuals age, the brain undergoes complex changes including neurodegeneration [6]. The two main neuropathological hallmarks are abnormal aggregates of amyloid and tau proteins, amyloid plaques, and neurofibrillary tangles, respectively [[6], [7], [8], [9], [10]]. The accumulation of these proteins may disrupt the communication between neurons, leading to neurodegeneration and cell death, which in turn causes cognitive decline.

Under abnormal conditions such as AD, tau becomes excessively phosphorylated, leading to a decrease in its affinity for microtubules. As a result, soluble hyperphosphorylated tau forms pathological soluble and insoluble aggregates known as neurofibrillary tangles (NFTs), which are hallmarks of AD. It is important to note that cognitive decline is more strongly correlated with tau pathology than amyloid beta (Aβ) plaques [11]. Currently, there is no cure for AD, and treatment primarily focuses on managing symptoms and slowing the disease progression. Medications such as cholinesterase inhibitors aim to increase the levels of essential neurotransmitters and temporarily improve memory and cognition. In addition to medication, non-pharmacological interventions such as engaging in cognitive activities, maintaining physical fitness, and following a healthy diet may also be beneficial. These interventions may offer some protection and help manage the symptoms [9]. Recent research has indicated that in older adults, a healthy lifestyle may provide a cognitive reserve to maintain cognitive abilities independent of the common neuropathologies of dementia [12]. RNA-Seq has revolutionized AD research by offering several advantages over previous approaches, making it a crucial tool for advancing our understanding of the disease. RNA-seq provides a more comprehensive and unbiased view of the transcriptome than traditional methods such as microarrays. It offers higher sensitivity, accuracy, and repeatability while producing lower noise [13]. This allows researchers to detect a wide range of transcripts, including novel and low-abundance transcripts, that may be critical for understanding AD pathogenesis. RNA-seq also enables identification of differentially expressed genes, alternative splicing events, and discovery of new lncRNAs and miRNAs involved in AD [13,14]. The application of RNA-Seq in AD research has led to the discovery of novel biomarkers, uncovering disease-associated pathways and elucidating the role of non-neuronal cells, such as microglia and astrocytes, in AD pathogenesis [14]. This level of detail is not possible using previous approaches, and it is necessary to advance our understanding of the complex mechanisms underlying AD. Thus, high-throughput RNA sequencing is necessary for AD research because it provides a more comprehensive, accurate, and detailed view of the transcriptome at both tissue and cellular levels. This technology enables researchers to gain new insights into AD pathology, identify potential therapeutic targets, and ultimately contribute to the development of more effective treatments for this devastating neurodegenerative disease [14]. Given that this disease primarily affects elderly individuals, it is essential to understand the relationship between aging and AD. In this study, we analyzed high-throughput RNA-seq data obtained from the brain tissues of elderly individuals with AD and compared them to those of aged but healthy individuals.

Using cutting-edge knowledge discovery tools, this study aimed to identify subtle changes in molecular pathways, gene ontologies, and potential drugs that could reverse AD-related gene signatures and pathologies, which are critical in the fight against this debilitating disease. The insights gained from this research may not only advance our understanding of AD pathogenesis, but also inform the development of novel diagnostic tools and therapeutic strategies, ultimately improving the lives of millions affected by this disease worldwide.

## 2 Materials and methods

### 2.1 Ethical statement

This research project was exempt from Institutional Review Board (IRB) approval because it did not employ animal models or human subjects. This study relied on RNA-seq datasets obtained from the Gene Expression Omnibus (GEO) [8,[15], [16]]. The use of the GEO dataset for in silico studies does not require additional IRB approval because the data are already de-identified and publicly available. Original ethical considerations were addressed during the initial data submission process [17]. This allowed us to freely access and analyze the dataset to generate new hypotheses, perform meta-analyses, and advance scientific knowledge, without the need for further ethical review [16].

### 2.2 Data source

We obtained high-throughput RNA-seq data from the GEO database (accession number GSE104704). These data were collected from healthy and diseased human brains, including eight young healthy brains, 10 aged healthy brains, and 12 aged diseased brains [17]. GEO RNA-seq Experiments Interactive Navigator (GREIN) software was used to obtain raw gene-level counts and filtered metadata for this dataset as previously described [18].

### 2.3 High-Dimensional data analysis using GREIN

GREIN is an open-source tool designed to reutilize GEO RNA-seq data [18]. The resource can be accessed at <https://shiny.ilincs.org/grein>, with the source code available at <https://github.com/uc-bd2k/grein> and the Docker container available at <https://hub.docker.com/r/ucbd2k/grein> (Access Date: December 22, 2023). GREIN offers a user-friendly interface for managing RNA-seq data and associated metadata, including quality control measures and expression pattern visualization. In the present study, we utilized GREIN to obtain raw counts of gene-level data and corresponding filtered metadata for further downstream analysis.

### 2.4 ExpressAnalyst analysis

Raw gene-level data and metadata from the GSE104704 dataset obtained from the GREIN database were further filtered and normalized using ExpressAnalyst (www.expressanalyst.ca). It was used for differential expression analysis, pathway enrichment, and visualization of the gene expression data. This tool was selected for its capacity to process RNA-seq data and perform a comprehensive gene expression analysis. The normalized data were subsequently used as inputs for the limma-based differential expression analysis method, which employs a log2-counts per million (log CPM) transformation and applied filters, such as the low abundance (cutoff value set at 4) and variance filters, as well as a filter for unannotated genes (cutoff value set at 15) [19,20] (Access Date: Jan 28, 2024). The resulting differentially expressed genes (DEGs) were further filtered based on a 1.5-fold cutoff and a P value less than or equal to 0.05. In gene expression studies, a lower threshold of ±1.5-fold or below with a P-value cut-off ≤0.05 is commonly used to identify DEGs [8,21,22]. The R/Bioconductor software package Limma, which is extensively used for analyzing gene expression data, including RNA-seq experiments, offers a comprehensive approach for DEG identification. This package was adapted for RNA-seq data analysis, and a linear model approach was utilized for statistical evaluation. Limma is particularly notable for its ability to handle intricate experimental designs and address the limitations associated with small sample sizes using information-borrowing techniques. For RNA-seq data analysis, Limma incorporated the voom transformation method, which converts count data to log-CPM values, facilitating the application of linear modeling and empirical Bayes methodologies [23,24]. Express Analyst was used for visualization, analysis, and statistical analysis of the gene expression data. Using the "Analysis Overview" section, visual analytics such as principal component analysis (PCA), enrichment networks based on the Kyoto Encyclopedia of Genes and Genomes (KEGG) and Reactome pathways, ridgeline charts, and heatmaps were derived to identify important features, functions, and correlations [19,20].

### 2.5 X2K web and Appyter analyses

The X2K web tool was used to infer upstream regulatory networks from DEGs [25]. This tool integrates transcription factor enrichment analysis, protein-protein interaction network expansion, and kinase enrichment analysis. Enrichment analysis was performed to predict transcription factors and kinases that regulate the expression of DEGs in elderly individuals with AD. X2K Appyter, a collection of web-based software applications (Expression2Kinases), predicts upstream regulatory networks associated with user-defined sets of genes (<https://appyters.maayanlab.cloud/#/X2K_Appyter>). Initially, discrete query gene sets were compared to ChEA3 libraries of transcription factor target gene sets, and protein interactors were compared to the KEA3 background database to predict kinase-substrate interactions, kinase–protein interactions, and interactions supported by co-expression and co-occurrence data to determine the upstream regulators likely responsible for DEGs in elderly patients with AD.

### 2.6 WebGestalt analysis

DEGs derived from elderly individuals with AD were analyzed using the WebGestalt tool (wGSEA) [26], an open-source software platform (<https://www.webgestalt.org/>) specifically designed for gene set enrichment analysis (GSEA) of *Homo sapiens*. It was used for Gene Ontology (GO) enrichment analysis, providing insights into the biological processes, molecular functions, and cellular components affected in AD. Gene Ontology (GO) terms, such as molecular function (MF), biological process (BP), and cellular component (CC) terms, were selected for each type of analysis. The reference list for each analysis included all mapped gene symbols from the selected platform genome, with the parameters for the enrichment analysis set at a minimum of 5 and a maximum of 2000 IDs in the category, a false discovery rate (FDR) of P < 0.05, computed using the Benjamini–Hochberg (BH) method, and the significance level of the top 10, as previously described [27].

### 2.7 Preranked gene set enrichment analysis

GSEA against a ranked list of genes using the preranked method was performed as described [28]. It was used to identify significantly enriched gene sets and pathways, thereby providing a broader understanding of the biological processes involved in AD. To facilitate comparisons between individuals with AD and healthy individuals, RNK-formatted files were generated based on the ranking metric log2Fc of DEGs. Gene matrix files (GMTs) were generated using gene signatures for significantly enriched (P < 0.05) biological processes, cellular components, and molecular functions obtained from WebGestalt analysis as previously described [28]. The GSEA pre-ranked analysis was performed by weighting each gene's contribution to the enrichment score according to the value of its ranking metric against GMT files using the Java-based desktop application GSEA 4.3.2 (Broad Institute, USA), with default settings as previously described [28].

### 2.8 L1000FWD and L1000CDS^2^ analyses

DEGs were analyzed using the L1000 Fire Works Display (L1000FWD) analysis [8,29,30] to identify the top 25 drugs and natural products with the potential to reverse AD-associated gene signatures in elderly individuals. Similarly, the same set of DEGs was analyzed using the L1000 Characteristic Direction Signature Search Engine (L1000CDS2) to identify the top 25 drugs and natural products with the potential to reverse AD-associated gene signatures in elderly individuals [8,31]. These tools have been used to identify potential drugs and natural products that could reverse AD-associated gene signatures. These tools leverage large-scale gene expression data to identify novel compounds with therapeutic potential.

## 3 Results

In the present study, we conducted an extensive analysis of next-generation sequencing data from GEO dataset GSE104704 to gain insights into the genetic basis of age-related AD. Specifically, we compared the gene-level expression data of individuals with AD to those of healthy individuals of the same age. Utilizing the ExpressAnalyst tool and filtered metadata and raw counts from GREIN, we identified 1255 DEGs between the two groups (aged, deceased vs. aged, healthy), as depicted in the volcano plot (Fig. 1A) (Supplementary File 1). PCA of the study group based on the DEGs (Fig. 1B) revealed distinct separation of the samples. Additionally, we generated a heatmap (Fig. 1C) depicting the top 250 DEGs ranked by adjusted p-values and Euclidean distances for comparison samples. Although the microtubule-associated protein tau (MAPT) gene was not differentially regulated (log2Fc = − 0.16; P < 0.08), we observed that MAPT antisense RNA 1 (log2Fc = 0.49; P < 0.025) and MAPT-intronic transcript 1 (MAPT-IT1) (log2Fc = 0.7; P < 0.006) were upregulated in individuals with AD compared with healthy individuals of the same age.

**Fig. 1:**
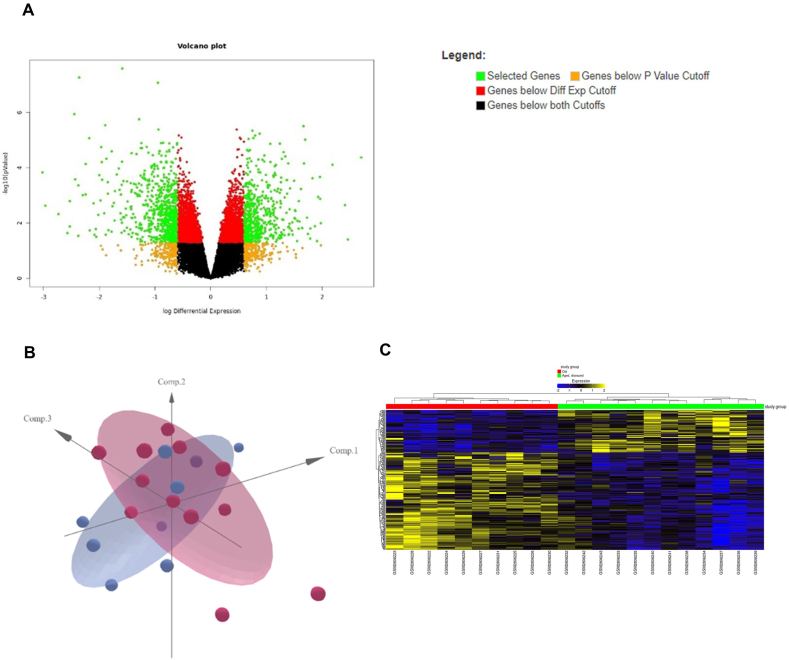
(A) Volcano plot of the 1255 differentially expressed genes (DEGs) associated with aged, disease vs aged, healthy determined using the Express Analyst tool. (B) Principal component analysis (PCA) of the study group based on differentially expressed genes (DEGs). (C) Heatmap across comparison samples based on the top 250 DEGs derived from the study group (aged, disease vs aged, healthy), ranked by adjusted p values and Euclidean distances.

Based on our observations, the levels of amyloid beta precursor protein (APP; log2Fc = −0.4; P < 0.15) and its interacting partners, such as amyloid beta precursor protein-binding family A member 1 (APBA1; log2Fc = −0.14; P < 0.35), amyloid beta precursor protein-binding family B member 1 (APBB1; log2Fc = −0.22; P < 0.10), APBB3 (log2Fc = −0.20, P < 0.13), amyloid beta precursor protein-binding family A member 3 (APBA3; log2Fc = 0.40; P < 0.056), amyloid beta precursor protein-binding protein 2 (APPBP2; log2Fc = 0.03; P < 0.28), and amyloid beta precursor protein-binding family B member 2 (APBB2; log2FC = 0.18; P < 0.31), were not significantly different from those of aged and healthy individuals. In contrast, the levels of APBB1, presenilin enhancer, and gamma-secretase subunit (PSENEN) were significantly higher in young individuals than those in old, aged, and old healthy individuals (Figure S1A and S1B). Furthermore, pre-ranked GSEA revealed negative enrichment of 312 protein-binding partners (Figure S1C) obtained from the Human Protein Atlas (Figure S1D) in elderly patients with AD. Among the top DEGs, we identified VGF nerve growth factor inducible (VGF), neuronal differentiation 6 (NEUROD6), potassium voltage-gated channel modifier subfamily F member 1 (KCNF1), potassium voltage-gated channel subfamily E regulatory subunit 5 (KCNE5), rabphilin 3A (RPH3A), corticotropin-releasing hormone (CRH), and MAPT intronic transcript 1 (MAPT-IT1) (Figure S1E–S1K).

**Fig. 2:**
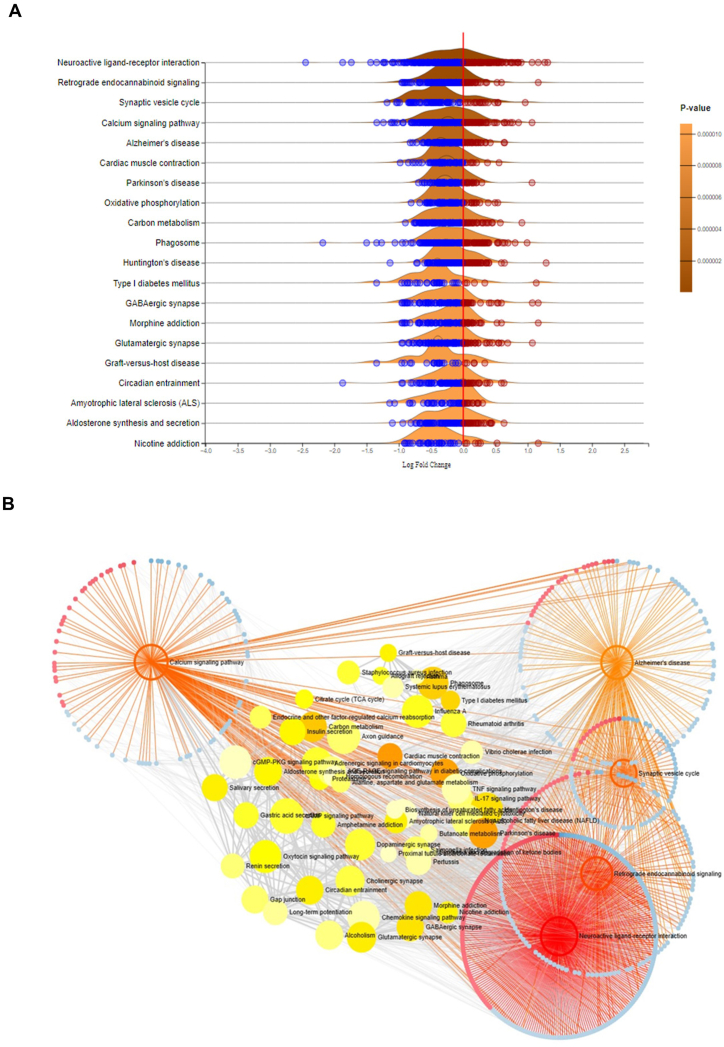
Differentially Regulated Kyoto Encyclopedia of Genes and Genomes (KEGG) Pathways in Elderly Patients with Alzheimer's Disease. (A) The ridgeline plot shows the results of the gene set enrichment analysis, where *p* values were calculated based on the GSEA algorithm implemented in the fgsea R package in Expression Analyst. (B) Network analysis of differentially regulated KEGG pathways showing the downregulated DEGs in key pathways, such as neuroactive receptor-ligand interaction, Alzheimer's disease, retrograde endocannabinoid signaling, the calcium pathway, the synaptic vesicle cycle, and other pathways, in elderly patients with AD.

DEGs were thoroughly analyzed using the Express Analyst tool to identify pathways that were differentially regulated in elderly individuals with AD. KEGG pathways, including neuroactive receptor-ligand interaction, Alzheimer's disease, retrograde endocannabinoid signaling, calcium pathway, synaptic vesicle cycle, and other pathways related to neurodegeneration, were significantly downregulated (P < 0.05) in elderly individuals with AD (Table S1). The results of the gene set enrichment analysis are illustrated in the ridgeline plot, which displays the p-values calculated using the GSEA algorithm, as implemented in the fgsea R package in ExpressAnalyst (Fig. 2A). Furthermore, network analysis of DEGs in KEGG pathways revealed downregulation of various genes (Fig. 2B) involved in KEGG pathways that are commonly affected in elderly individuals with AD.

**Fig. 3:**
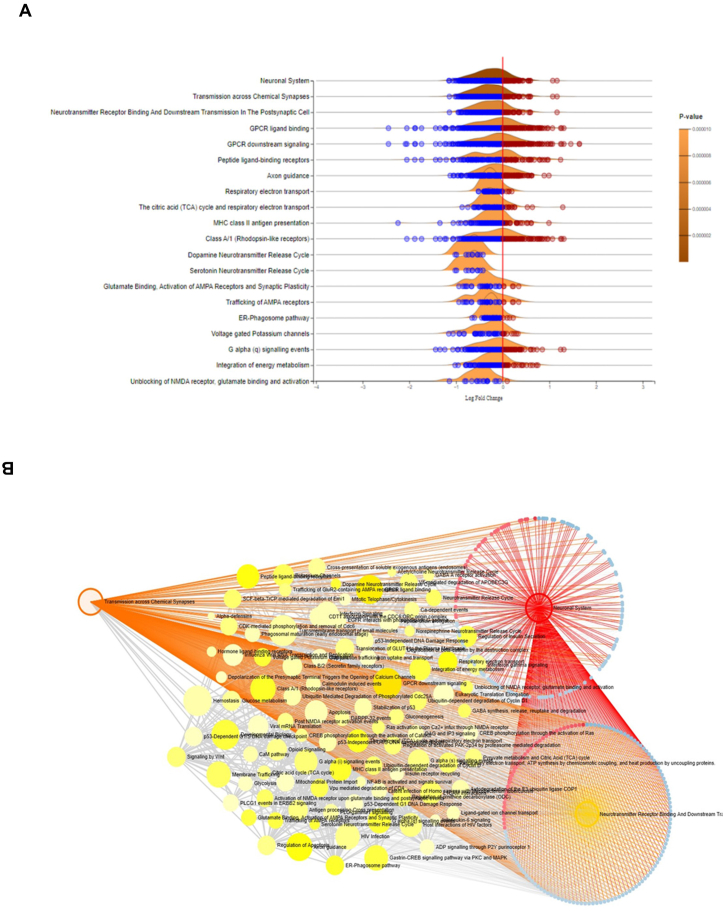
Differentially Regulated Reactome Pathways in Elderly Patients with Alzheimer's Disease. (A) The ridgeline plot shows the results of the gene set enrichment analysis, where *p* values were calculated based on the GSEA algorithm implemented in the fgsea R package in Expression Analyst. (B) Network analysis of differentially regulated Reactome pathways showing the downregulated expression of DEGs in key pathways, such as the neuronal system, transmission across chemical synapses, neurotransmitter receptor binding, and downstream transmission in postsynaptic cells, and other pathways in elderly patients with AD.

The Reactome pathways, which included the Neuronal System, Transmission across Chemical Synapses, Neurotransmitter Receptor Binding and Downstream Transmission in Postsynaptic Cells, and other pathways related to brain function, were significantly downregulated (P < 0.05) in elderly individuals with AD (Table S2). The results of the gene set enrichment analysis are depicted in a ridgeline plot, which displays the p-values calculated using the GSEA algorithm, as implemented in the fgsea R package in ExpressAnalyst (Fig. 3A). Furthermore, network analysis of the DEGs in the Reactome pathways revealed the downregulation of a variety of genes (Fig. 3B) involved in the reactome pathways that are known to be affected in elderly individuals with AD.

**Fig. 4:**
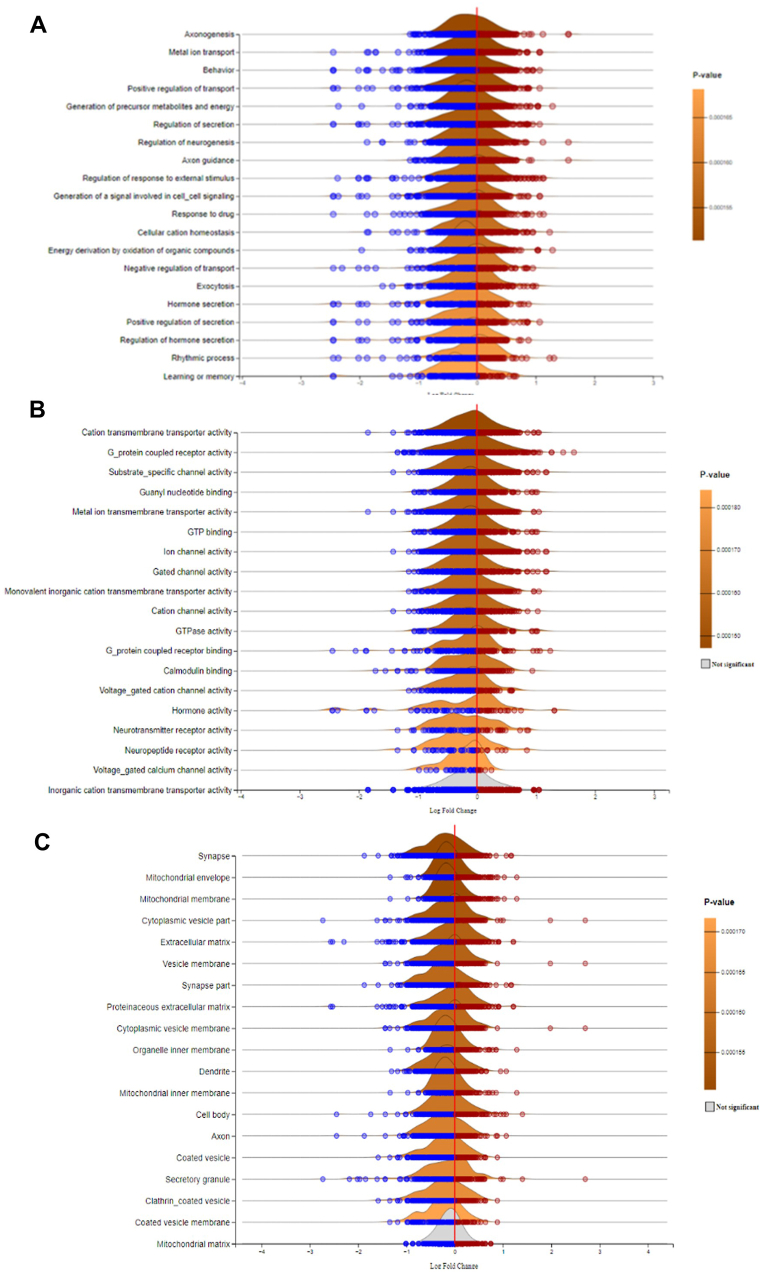
Differentially Regulated Gene Ontologies (GO) in Elderly Patients with Alzheimer's Disease. (A) Ridgeline plot showing the differentially regulated biological process (BP), (B) molecular function (MF), and (C) cellular component (CC) terms based on gene set enrichment analysis (GSEA), where *p* values were calculated based on the GSEA algorithm implemented in the fgsea R package.

ExpressAnalyst was instrumental in determining the GOs that was differentially regulated in elderly individuals with AD, specifically in terms of BP, MF, and CC (Fig. 4). Our analysis revealed that several BP categories, including axonogenesis, metal ion transport, behavior, positive regulation of transport, generation of precursor metabolites and energy, regulation of secretion, regulation of neurogenesis, axon guidance, regulation of response to external stimulus, response to drug, generation of a signal involved in cell-to-cell signaling, cellular cation homeostasis, energy derivation by oxidation of organic compounds, negative regulation of transport, exocytosis, hormone secretion, positive regulation of secretion, regulation of hormone secretion, rhythmic process, learning or memory, cellular respiration, synapse organization, regulation of neurotransmitter levels, response to carbohydrate stimulus, neurotransmitter secretion, feeding behavior, glutamate receptor signaling pathway, aerobic respiration, response to biotic stimulus, and positive regulation of cellular component organization were significantly affected (P < 0.01) in elderly individuals with AD (Table S3).

Our analysis revealed that many molecular functions, such as cation transmembrane transporter activity, G-protein coupled receptor activity, inorganic cation transmembrane transporter activity, guanyl nucleotide binding, substrate-specific channel activity, metal ion transmembrane transporter activity, GTP binding, ion channel activity, gated channel activity, monovalent inorganic cation transmembrane transporter activity, cation channel activity, GTPase activity, G-protein coupled receptor binding, calmodulin binding, voltage-gated cation channel activity, hormone activity, neurotransmitter receptor activity, neuropeptide receptor activity, voltage-gated calcium channel activity, delayed rectifier potassium channel activity, chemokine receptor binding, chemokine activity, neuropeptide hormone activity, voltage-gated channel activity, cytokine activity, calcium ion transmembrane transporter activity, voltage-gated potassium channel activity, structural constituents of the cytoskeleton, calcium channel activity, and cytokine receptor binding were significantly affected in elderly individuals with AD (P < 0.01, Table S4).

Our analysis uncovered that a variety of CC categories, including the mitochondrial envelope, synapse, mitochondrial membrane, cytoplasmic vesicle, extracellular matrix, vesicle, membrane, synapse part, proteinaceous extracellular matrix, cytoplasmic vesicle membrane, organelle inner membrane, dendrite, mitochondrial inner membrane, cell body, mitochondrial matrix, axon, coated vesicle, secretory granule, clathrin-coated vesicle, coated vesicle membrane, transport vesicle, mitochondrial membrane part, synaptic vesicle, growth cone, site of polarized growth, voltage-gated potassium channel complex, mitochondrial respiratory chain, mitochondrial respiratory chain complex I, NADH dehydrogenase complex, respiratory chain complex I, and cell surface, were significantly altered in elderly individuals with AD (P < 0.01) (Table S5). Additionally, we identified numerous downregulated BP and CC categories related to neurodegeneration and motifs (Figure S2) in the elderly individuals with AD (Tables S6–S8). These findings were supported by statistical analyses (Table S9). The list of DEGs obtained from ExpressAnalyst analysis and all supplementary tables (S1–S9) are provided in Supplementary File 1.

**Fig. 5:**
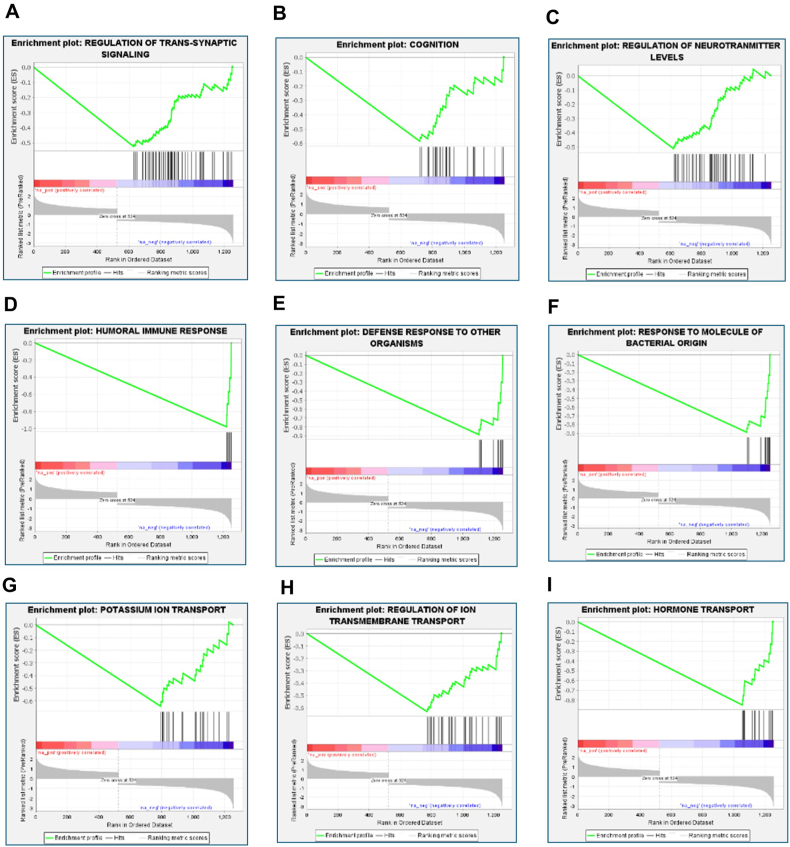
Preranked Gene Set Enrichment Analysis (GSEA) was performed using GSEA software (version 4.3.2; Broad Institute, USA) for Gene Ontology Biological Processes (GO BPs) obtained using WebGestalt analysis of DEGs in elderly patients with AD. Negative enrichment of GO BPs included (A) regulation of transsynaptic signaling, (B) cognition, (C) regulation of neurotransmitter levels, (D) humoral immune response, (E) defense response to other organisms, (F) response molecule of bacterial origin, (G) potassium ion transport, (H) regulation of ion transmembrane transport, and (I) Hormone transport in elderly patients with AD.

The GO BP information obtained via WebGestalt analysis of DEGs in elderly patients with AD was subjected to pre-ranked GSEA. The results revealed a negative enrichment of biological processes such as the regulation of transsynaptic signaling, cognition, regulation of neurotransmitter levels, humoral immune response, defense response to other organisms, responses to molecules of bacterial origin, potassium ion transport, regulation of ion transmembrane transport, and hormone transport in elderly patients with AD (Fig. 5). The gene signatures associated with GO BPs are provided in Supplementary File 2.

**Fig. 6:**
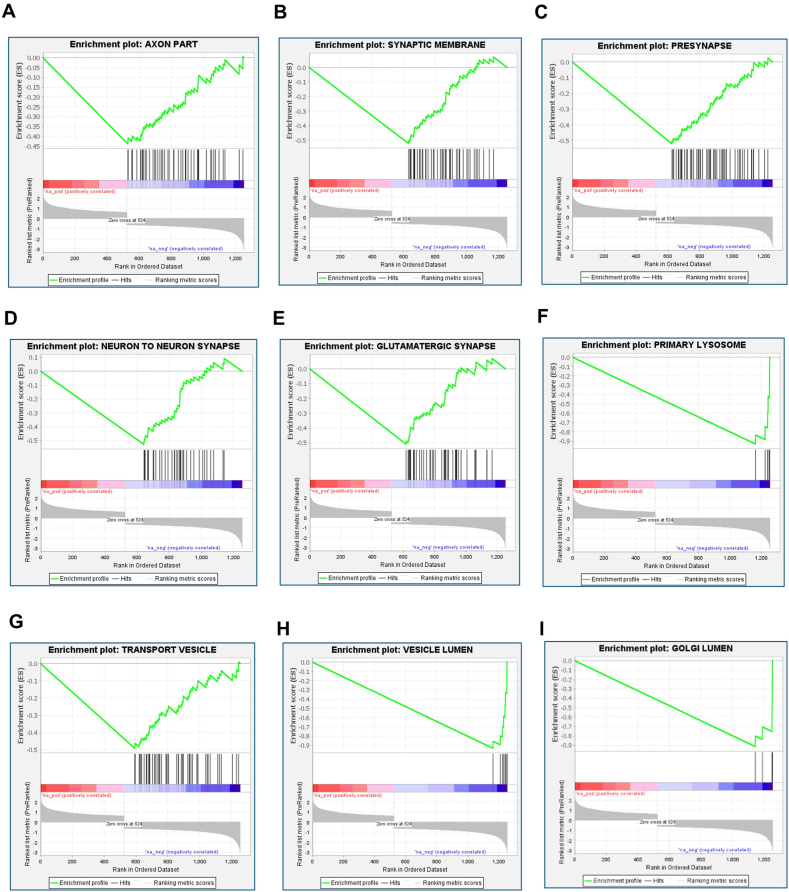
Preranked Gene Set Enrichment Analysis (GSEA) was performed using GSEA software (version 4.3.2; Broad Institute, USA) for Gene Ontology Cellular Component (GO CCs) obtained using WebGestalt analysis of DEGs in elderly patients with AD. Negative enrichment of GO CCs included (A) axon part (B) synaptic membrane, (C) presynapse, (D) neuron to neuron synapse, (E) glutamatergic synapse, (F) primary lysosome, (G) transport vesicle, (H) vesicle lumen and (I) Golgi lumen in elderly patients with AD.

Preranked GSEA was performed for the GO CCs obtained using WebGestalt analysis of DEGs in elderly patients with AD (Fig. 6). We observed negative enrichment of GO CCs, such as axons, synaptic membranes, presynapses, neuron-to-neuron synapses, glutamatergic synapses, primary lysosomes, transport vesicles, vesicle lumen, and Golgi lumen, in elderly patients with AD. The gene signatures associated with GO CCs are provided in Supplementary File 2.

**Fig. 7:**
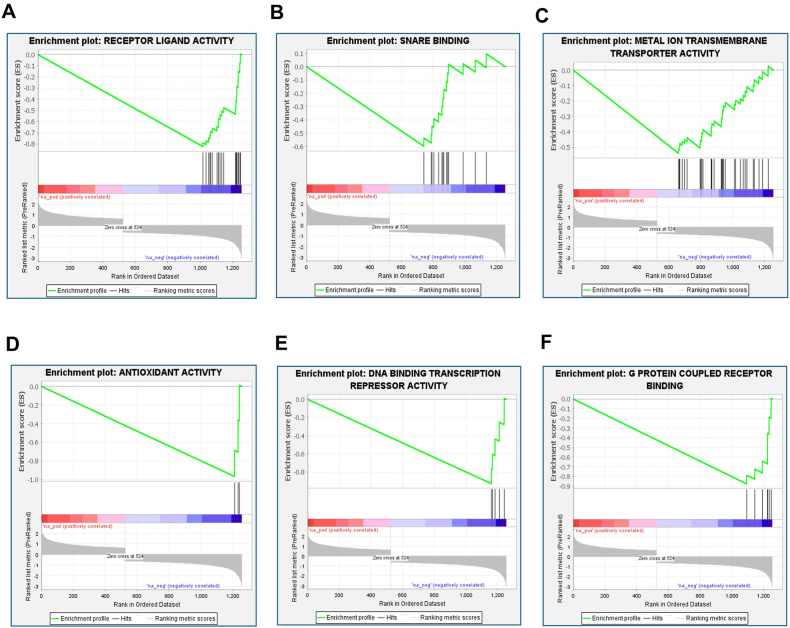
Preranked Gene Set Enrichment Analysis (GSEA) was performed using GSEA software (version 4.3.2; Broad Institute, USA) for Gene Ontology Molecular Function (GO MFs) information obtained via WebGestalt analysis of DEGs in elderly patients with AD. Negative enrichment of GO MFs included (A) receptor ligand activity, (B) SNARE binding, (C) metal ion transmembrane transporter activity, (D) antioxidant activity, (E) DNA binding transcription repressor activity, and (F) G protein-coupled receptor binding.

Similarly, a preranked GSEA was conducted for GO MF data obtained through WebGestalt analysis of DEGs in elderly patients with AD (Fig. 7). The results revealed negative enrichment for several GO MF categories, including receptor-ligand activity, SNARE binding, metal ion transmembrane transporter activity, antioxidant activity, DNA binding transcription repressor activity, and G protein-coupled receptor binding. The gene signatures associated with GO MF are provided in Supplementary File 2.

**Fig. 8:**
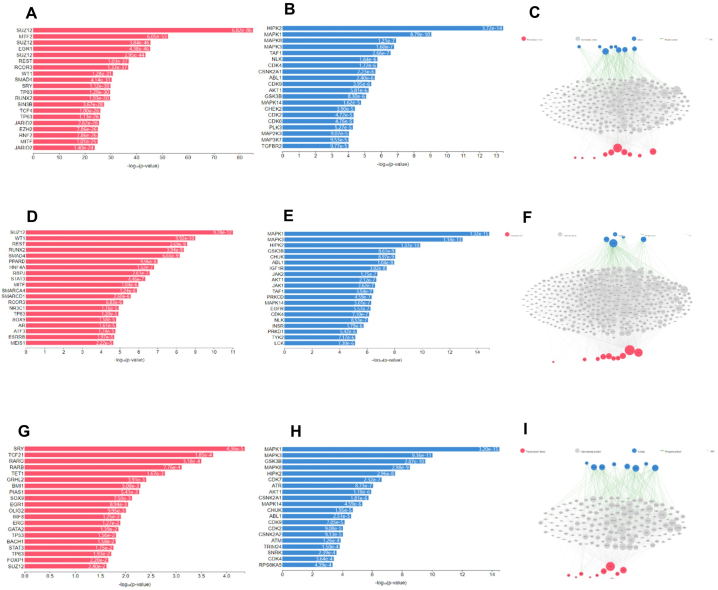
The X2K Web Tool Analysis of Upstream Regulator Networks. The X2K web tool was used to identify upstream regulatory networks from the gene signatures derived from elderly patients with AD. (A–C) Transcription factor enrichment analysis, kinase enrichment analysis, and protein-protein interaction networks were predicted to regulate the expression of the DEGs in elderly patients with AD. (D–F) Transcription factor enrichment analysis, kinase enrichment analysis, and protein-protein interaction networks were predicted to regulate the downregulated genes in elderly patients with AD. (G–I) Transcription factor enrichment analysis, kinase enrichment analysis, and protein-protein interaction networks were predicted to regulate the upregulated genes in elderly patients with AD.

We used the X2K web tool to infer upstream regulatory networks from gene signatures obtained from elderly individuals with AD. By integrating transcription factor enrichment analysis, protein-protein interaction network expansion, and kinase enrichment analysis, we generated networks of transcription factors, proteins, and kinases that were predicted to regulate the expression of both downregulated and upregulated genes in elderly individuals with AD. Our findings revealed that the top upstream transcription factors, including SUZ12, MTF2, REST, EGR1, and SRY, targeted 321, 214, 310, 612, and 175 DEGs (P < 0.01), respectively, in elderly individuals with AD (Fig. 8A). Additionally, the top upstream kinases, HIPK2, MAPK1, MAPK8, MAPK3, and TAF1, targeted 21, 41, 24, 32, 13, and 8 DEGs (P < 0.01), respectively, in elderly individuals with AD (Fig. 8B). The protein-protein interactions of transcription factors (TFs) and kinases and their respective protein targets are depicted in Fig. 8C. Furthermore, genes with downregulated expression were predicted via transcription factor enrichment analysis, kinase enrichment analysis, and protein-protein interaction network construction (Fig. 8G–I). Transcription factor enrichment, kinase enrichment, and protein-protein interaction network analyses predicted upregulated genes, providing additional insights into upstream regulatory TFs and kinases in elderly individuals with AD.

**Fig. 9:**
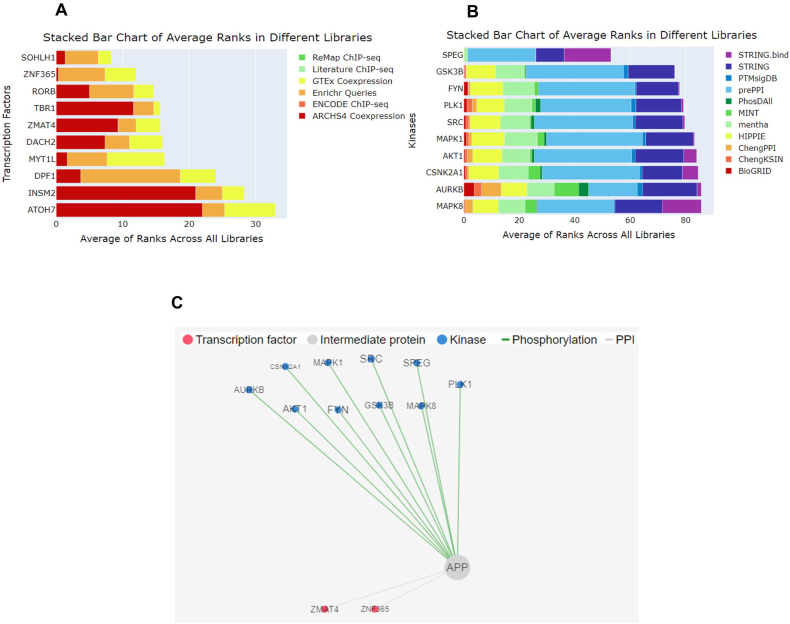
X2K Appyter Analysis (A) The bar chart is a mean rank bar chart of the highest-ranked transcription factors from ChEA3. The y-axis displays the different transcription factors, and the x-axis displays the average mean rank of a transcription factor across all the available libraries. The highest ranked transcription factors were SOHLH1, ZNF365, RORB, TBR1, ZMAT4, DACH2, MYT1L, DPF1, INSM2, and ATOH7 in elderly patients with AD. (B) The bar chart is a mean rank bar chart for the top-ranked kinases from KEA3, such as SPEG, GSK3B, FYN, PLK1, SRC, MAPK1, AKT1, CSNK2A1, AURKB, and MAPK8, in elderly patients with AD. The y-axis displays the different kinases, and the x-axis displays the mean rank of a specific kinase across all the available libraries. (C) X2K network visualization. The red nodes represent TFs, the white nodes represent intermediate proteins/transcription factor interactors, and the blue nodes represent kinases. After connecting the nodes, the white lines represent protein-protein interactions (PPIs) between TFs and their interactors, and the green lines represent the phosphorylation of kinases.

The X2K web tool analysis generated a list of top-ranked transcription factors across all libraries in elderly patients in the AD cohort (Fig. 9). The most influential factors are SOHLH1, ZNF365, RORB, TBR1, ZMAT4, DACH2, MYT1L, DPF1, INSM2, and ATOH7. Additionally, top-ranked kinases from KEA3 were observed in the elderly AD cohort, including SPEG, GSK3B, FYN, PLK1, SRC, MAPK1, AKT1, CSNK2A1, AURKB, and MAPK8. The X2K network visualization analysis depicted transcription factors (red nodes), intermediate protein/transcription factor interactors (white nodes), and kinases (blue nodes), with connecting lines indicating protein-protein interactions (PPIs) between factors and their interactors (white lines) and phosphorylation of kinases (green lines). In our study, only one relevant TF interactor, APP, was identified based on the results of X2K Appyter analysis.

**Fig. 10:**
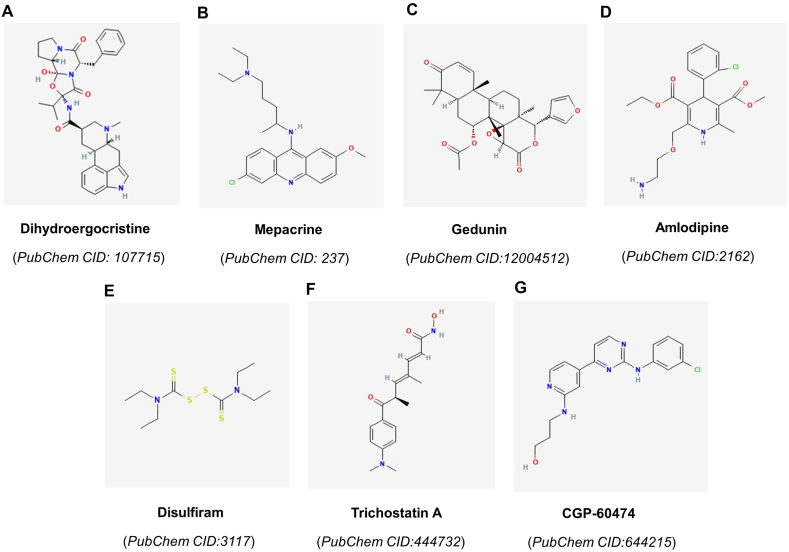
Potential anti-AD natural products or drugs (A) Dihydroergocristine, (B) Mepacrine (C) Gedunin, (D) Amlodipine, (E) Disulfiram, (F) Trichostatin, and (G) ACGP-60474 that reverse DEGs in elderly patients with AD compared with aged healthy individuals based on L1000FWD and L1000CDS^2^ analysis.

Based on the results obtained using the L1000FWD tool, we identified the top 25 drugs or natural products with the potential to reverse DEGs associated with elderly individuals diagnosed with AD. Our findings indicated that dihydroergocristine, mepacrine, gedunin, amlodipine, and disulfiram had significant effects (P < 0.01; Z score <1.75; combined score < −7.65) on the gene signatures derived from elderly individuals with AD (Table 1). Notably, these findings align with those of our previous report, in which we utilized the L1000CDS2 tool to identify the top 25 drugs or natural products, including trichostatin A and CGP-60474 (Fig. 10), that could reverse the DEGs associated with elderly individuals diagnosed with AD (Table 2).

**Table 1:** The top 25 drugs or natural products that reverse DEGs in elderly patients with AD based on L1000FWD analysis.

S.No	Signature ID	Drug or Natural product	Similarity Score	p-value	q-value	Z score	combined score
1	CPC005_A375_24H:BRD-A44701612-066-01-9:10	dihydroergocristine	−0.0442	1.07E-06	1.93E-03	1.91	−11.4
2	CPC001_VCAP_24H:BRD-A82656074-003-01-2:10	naltrindole	−0.0427	1.41E-05	1.22E-02	1.88	−9.13
3	CPC001_PC3_6H:BRD-K84421793-001-01-1:10	BRD-K84421793	−0.0474	2.13E-06	3.26E-03	1.86	−10.56
4	CPC006_HEPG2_6H:BRD-A45889380-300-04-8:10	mepacrine	−0.0458	4.19E-06	5.60E-03	1.84	−9.88
5	CPC005_A375_6H:BRD-A22713669-001-01-9:10	BVT-948	−0.0427	3.80E-05	2.43E-02	1.84	−8.14
6	CPC005_HA1E_24H:BRD-A49225603-045-05-0:10	alimemazine	−0.0395	1.46E-04	5.97E-02	1.84	−7.05
7	CPC007_A375_6H:BRD-K95196255-001-09-0:10	BRD-K95196255	−0.0379	1.56E-04	6.15E-02	1.84	−6.99
8	CPC005_VCAP_24H:BRD-A54845972-066-01-5:10	dihydroergotamine	−0.0411	4.28E-05	2.65E-02	1.83	−8.01
9	CPC005_MCF7_24H:BRD-K21350491-001-02-1:10	phenamil	−0.0395	9.65E-05	4.39E-02	1.83	−7.36
10	CPC006_HEPG2_6H:BRD-K00317371-001-02-0:10	RITA	−0.0395	2.64E-04	8.25E-02	1.83	−6.56
11	CPC004_VCAP_24H:BRD-A22032524-074-04-0:10	amlodipine	−0.0379	3.36E-04	9.46E-02	1.83	−6.37
12	CPC002_HCC515_24H:BRD-K21064560-001-01-6:10	BRD-K21064560	−0.0395	4.45E-04	1.13E-01	1.82	−6.1
13	CPC006_HCC515_6H:BRD-K92301463-001-03-0:10	16,16-dimethyl prostaglandin-e2	−0.049	5.53E-07	1.39E-03	1.81	−11.33
14	CPC004_HA1E_24H:BRD-A17819071-001-02-1:10	gedunin	−0.0427	5.83E-05	3.28E-02	1.81	−7.65
15	CPC006_HCT116_6H:BRD-K53903639-001-01-3:80	CHEMBL-1222381	−0.0458	4.19E-06	5.60E-03	1.8	−9.7
16	CPC006_HA1E_6H:BRD-K60623809-001-02-0:10	SU-11652	−0.0395	1.79E-04	6.78E-02	1.8	−6.74
17	CPC007_PC3_24H:BRD-K83194053-001-07-7:10	BRD-K83194053	−0.0474	6.67E-06	7.72E-03	1.77	−9.14
18	CPC006_A549_24H:BRD-K53903639-001-01-3:80	CHEMBL-1222381	−0.0395	4.32E-04	1.11E-01	1.77	−5.94
19	CPC012_HA1E_6H:BRD-K86269644-001-01-3:10	BRD-K86269644	−0.0442	1.22E-05	1.10E-02	1.76	−8.66
20	CPC008_MCF7_6H:BRD-A31946439-019-01-5:10	BRD-A31946439	−0.0395	2.25E-04	7.70E-02	1.76	−6.43
21	CPC007_VCAP_6H:BRD-K07668032-001-07-9:10	BRD-K07668032	−0.0379	8.29E-04	1.68E-01	1.76	−5.42
22	CPC011_MCF7_6H:BRD-K32744045-001-25-4:10	disulfiram	−0.0506	3.15E-07	9.63E-04	1.75	−11.41
23	CPC006_HT29_24H:BRD-K53903639-001-01-3:80	CHEMBL-1222381	−0.0458	3.06E-05	2.08E-02	1.75	−7.91
24	CPC011_PC3_24H:BRD-K15916496-001-23-8:10	clotrimazole	−0.0379	6.98E-04	1.51E-01	1.75	−5.54
25	CPC019_PC3_24H:BRD-K37043259-001-01-8:10	BRD-K37043259	−0.0458	8.49E-07	1.65E-03	1.74	−10.57

**Table 2:** Top 25 drugs or natural products that reversed DEGs in elderly patients with AD based on L1000CDS^2^ analysis.

Rank	Overlap	Perturbation	Cell-line	Dose	Time
1	0.0474	CGP-60474	HCC515	0.37 μm	24 h
2	0.0395	528116.cdx	MCF7	0.09 μm	24.0 h
3	0.0363	CGP-60474	HA1E	0.12 μm	24 h
4	0.0363	CGP-60474	HCC515	1.11 μm	24 h
5	0.0348	480743.cdx	A375	80.0 μm	24.0 h
6	0.0348	Emetine Dihydrochloride Hydrate (74)	MCF7	0.63 μm	6.0 h
7	0.0348	BRD-K77908580	MCF7	10.0 μm	24.0 h
8	0.0348	F1566-0341	MCF7	10.0 μm	6.0 h
9	0.0348	JNK-9 L	HCC515	10 μm	24 h
10	0.0348	CGP-60474	HCC515	1.11 μm	24 h
11	0.0332	TENIPOSIDE	A375	10.0 μm	6.0 h
12	0.0332	480743.cdx	HA1E	80.0 μm	24.0 h
13	0.0332	QUINACRINE HYDROCHLORIDE	HT29	10.0 μm	24.0 h
14	0.0332	trichostatin A	HEPG2	10.0 μm	6.0 h
15	0.0332	BRD-K91145395	MCF7	10.0 μm	6.0 h
16	0.0332	WZ-3105	A549	1.11 μm	24 h
17	0.0332	CGP-60474	A375	1.11 μm	24 h
18	0.0332	CGP-60474	A375	0.04 μm	24 h
19	0.0316	trichostatin A	HT29	10.0 μm	24.0 h
20	0.0316	phorbol-12-myristate-13-acetate (PMA)	MCF7	10.0 μm	24.0 h
21	0.0316	manumycin A	SNUC5	10.0 μm	6.0 h
22	0.0316	BRD-K39987650	MCF7	10.0 μm	24.0 h
23	0.0316	trichostatin A	MCF7	10.0 μm	24.0 h
24	0.0316	BRD-K12867552	MCF7	10.0 μm	24.0 h
25	0.0316	CGP-60474	HCC515	0.37 μm	24 h

## 4 Discussion

The current study involved a comprehensive analysis of AD in elderly individuals using the latest RNA-seq data and advanced NGKD methods to elucidate the intricate mechanisms of pathways, gene ontologies, and transcription factors that regulate the complex networks of proteins. Ultimately, our goal was to identify potential therapies for dementia. As individuals age, their brains undergo complex changes, including neurodegeneration, making age the most significant risk factor for AD [6]. AD is characterized by two main neuropathological hallmarks: accumulation of abnormal aggregates of amyloid or tau proteins, known as amyloid plaques, and neurofibrillary tangles [[6], [7], [8], [9]]. The accumulation of these proteins may disrupt the communication between neurons, leading to neurodegeneration and cell death, which in turn causes cognitive decline. The quest for effective treatments for AD is both challenging and rapidly evolving, and encompasses a broad range of potential candidates. Thus, exploring the complex molecular machinery involved in elderly individuals with AD and some drugs and natural products that can reverse AD-associated gene signatures using cutting-edge knowledge discovery tools is advantageous [8].

We used the GREIN platform, which is a user-friendly graphical user interface for managing RNA-seq data (counts) and the associated metadata [18]. This platform also includes quality control measures, expression pattern visualization, an entire dataset, sample size analysis, and power analysis [18]. To identify subtle differences in gene expression signatures between elderly individuals with AD and aged healthy controls, we derived DEGs with a 1.5-fold cutoff and a p-value of 0.05 using RNA-seq data (counts) and associated metadata as input files in the ExpressAnalyst tool [19,20].

Our study revealed that neuroactive receptor-ligand interactions, the synaptic vesicle cycle, the neuronal system, transmission across chemical synapses, neurotransmitter receptor binding, and downstream transmission in postsynaptic cell pathways, which are vital for normal brain function, are affected by AD in elderly individuals. These findings align with those of recent studies that have demonstrated that synapses are vulnerable and critical targets in AD. Synapse loss is now considered to be one of the primary biological correlates of cognitive decline in AD, occurring before neuronal loss. Ample evidence suggests that synaptic dysfunction precedes this loss, supporting the notion that synaptic failure is a critical stage of disease pathogenesis. Accumulation of amyloid or tau proteins, two hallmark features of AD, has been shown to have detrimental effects on synaptic function in both animal and cellular models of this disease. Furthermore, evidence suggests that these two proteins may exert synergistic effects on neurophysiological dysfunctions. However, unexpectedly, no significant difference was observed in the expression of genes, such as MAPT and APP, in elderly individuals with AD compared to that in aged but healthy individuals. Most of the protein binding partners of APP were downregulated in elderly individuals with AD, but the upregulation of MAPT antisense RNA 1 and MAPT-IT1 in elderly individuals with AD suggests that there is a counterregulatory mechanism in the brains of elderly individuals with AD that regulates MAPT and its interaction with APP. A recent study showed that the use of synthetic, externally administered tau-targeting antisense oligonucleotides (MAPTRx) in patients with mild AD significantly reduced tau levels in cerebrospinal fluid (CSF) [32,33]. Natural antisense transcripts (NATs) of the MAPT gene, which encodes the tau protein, have been identified and may play a role in regulating MAPT expression in AD [32]. The presence of NATs in MAPT suggests a potential mechanism for transcriptional regulation of this important AD-associated gene. Interestingly, although MAPT NATs have been detected, their specific functions and effects on AD pathology remain unclear. The identification of NATs for multiple AD-associated genes, including APP, BACE2, and APH1A, in addition to MAPT, highlights the potential role of antisense transcripts in modulating the expression of genes involved in AD pathogenesis [34]. Thus, the discovery of MAPT antisense transcripts provides insight into an additional layer of regulation of this key AD-related gene. Further research is needed to determine the precise mechanisms by which MAPT NATs influence tau expression and contribute to AD risk and progression. Understanding the role of antisense transcripts could lead to novel therapeutic strategies targeting MAPT regulation in AD. APP has two homologs, APLP1 and APLP2, which exhibit functional redundancy [35]. A decrease in the expression of APP, APLP1, APLP2, and their various binding partners, such as APBB1 and PSENEN, in elderly individuals with AD compared with aged but healthy individuals may contribute to the negative enrichment of pathways and gene ontologies related to neurodegeneration and AD because the APP gene encodes a precursor protein that generates various peptides after cleavage by secretases. Some of these peptides are secreted and activate transcription by interacting with the acetyltransferase complex, APBB1/TIP60 [36]. Two of these peptides have antimicrobial properties, which may partly explain the negative enrichment of genes related to humoral immune response against bacteria, fungi, and other microorganisms in elderly individuals with AD [37]. APP functions as a cell surface receptor on neurons, promoting neurite growth, neuronal adhesion, and axonogenesis. It also plays a role in synaptogenesis by interacting with neighboring cells. Additionally, APP is involved in cell mobility, transcriptional regulation, protein-protein interactions, and apoptosis-inducing pathways. Furthermore, it mediates the axonal transport of beta-secretase and presenilin 1, which participate in the anterograde transport of cargo toward synapses in axons [38].

The VGF gene, which is specifically expressed in a subpopulation of neuroendocrine cells and upregulated by nerve growth factors, has unknown functions related to neurons. Its expression is downregulated in elderly AD patients [[39], [40], [41]]. Voltage-gated potassium channels, which play a crucial role in regulating various physiological processes such as neurotransmitter release, heart rate, insulin secretion, and cellular function, are highly complex and further categorized into subfamily F, which is encoded by the KCNF1 gene. KCNE5 functions in conjunction with voltage-gated potassium channels and modulates ion selectivity, voltage dependence, and plasma membrane recycling [[42], [43], [44]]. RPH3A, which is involved in neurotransmitter release and synaptic vesicle trafficking, functions as an effector of the small G protein RAB3A during late-stage neurotransmitter exocytosis [45,46]. The NEUROD6 family of basic helix-loop-helix transcription factors is involved in the development and differentiation of the nervous system [47], whereas the CRH pre-proprotein is proteolytically processed to generate mature neuropeptide hormones and subsequently induces the release of adrenocorticotropic hormone from the pituitary gland. A marked reduction in the levels of this protein has been observed in association with AD [48,49].

Downregulation of genes such as VGF, NEUROD6, and RPH3A has been implicated in the pathogenesis of AD. VGF is a neurotrophic factor that plays critical roles in neuronal survival and synaptic plasticity. Reduced gene expression in AD may contribute to neuronal loss and cognitive decline [[39], [40], [41]]. Collectively, the downregulation of these genes suggests a complex interplay between the molecular mechanisms that contribute to the pathogenesis of AD. Reduced expression of VGF may result in diminished neuroprotection, whereas downregulation of NEUROD6 may lead to increased neuronal vulnerability. Concurrently, a decrease in RPH3A levels may exacerbate synaptic dysfunction, which is a hallmark of AD. These findings provide a more comprehensive understanding of the links between gene dysregulation, progressive neurodegeneration, and cognitive impairment observed in AD, highlighting potential targets for therapeutic interventions. Therefore, the downregulation of VGF, NEUROD6, KCNF1, KCNE5, CRH, and RPH3A may contribute to the cognitive decline observed in elderly individuals with AD.

Negative enrichment of genes related to primary lysosomes, transport vesicles, vesicle lumen, and Golgi lumen has been observed in elderly individuals with AD. Studies have implicated genes that regulate lysosomal function in common sporadic neurodegenerative diseases including AD [50,51]. Proper lysosomal function is essential for maintenance of the nervous system [52]. Furthermore, negative enrichment of genes associated with potassium ion transport and regulation of ion transmembrane transport has been observed in elderly individuals with AD. Abnormal functioning of potassium ion channels has been identified in platelets of patients with AD [53]. Potassium (K+) channels, which have more than 80 subunits, are classified as ion channels and exhibit significant diversity and ubiquity. These channels play a critical role in regulating membrane polarization and cell excitability in the brain and other tissues and in controlling the movement of both intracellular and extracellular K+, which impacts neurotransmitter release. Dysfunctional K+ channels in neurons are associated with a diverse range of neurological disorders, particularly AD [54].

Previous studies have demonstrated that both APP and tau are regulated by REST or SUZ12, and dysregulated plasticity proteins are associated with SUZ12 and REST signaling, suggesting aberrant gene repression in AD [55,56]. In our study, the majority of downregulated genes in elderly individuals with AD were primarily regulated by the transcription factors SUZ12 and REST, with an increase in mitogen-activated protein kinase (MAPK) signaling. A recent study on molecular processes and transcription factor enrichment analysis suggested that the increased expression of neuroplasticity proteins detected in the AD group with elevated total tau (t-tau) could be attributed to an increase in mitogenic MAPK signaling or a decrease in gene repression by REST and SUZ12 [[57], [58]]. Earlier postmortem AD studies and iPSC neurons from individuals with sporadic AD have shown derepression of gene expression by REST/SUZ12 [55,[56], [58]]. According to the X2K aptamer prediction, top-ranked kinases, such as SPEG, GSK3B, FYN, PLK1, SRC, MAPK1, AKT1, CSNK2A1, AURKB, and MAPK8, interact with APP in elderly individuals with AD. Interestingly, APP is the only relevant TF that interacts with at least two of the top transcription factors, ZNF365 and ZMAT4.

Currently approved drugs, such as cholinesterase inhibitors (donepezil, rivastigmine, and galantamine), aim to prevent the degradation of acetylcholine, a neurotransmitter crucial for memory and cognitive function. These drugs do not halt disease progression but can modestly enhance cognitive function and activities of daily living in the early and moderate stages of AD [59]. Memantine, which targets NMDA receptors involved in learning and memory, can help maintain cognitive function and slow symptom progression in patients with moderate AD [60]. In terms of emerging therapies, monoclonal antibodies, such as aducanumab and lecanemab, are laboratory-generated molecules that focus on amyloid plaques, a hallmark of AD pathology. Although their effectiveness and safety are still debated, these therapies have been approved for use in early-stage AD [61]. Based on NGKD tools, the present study revealed that dihydroergocristine (DHEC), mepacrine, gedunin, amlodipine, and disulfiram may reverse the gene signatures associated with AD in elderly individuals. The transition from established treatments to novel candidates identified using NGKD may lead to a promising shift in AD research and drug development. DHEC mesylate, a component of ergoid mesylates, has shown potential as a novel therapeutic agent for AD. Studies have demonstrated that DHEC mesylate administration alleviates spatial memory disorders and Alzheimer-type pathologies [62]. It improves aberrant bisecting N-glycosylation, a potential AD biomarker, and protects against AD via the AMPK and ERK signaling pathways [62]. Additionally, DHEC has been identified as a direct inhibitor of γ-secretase, reducing amyloid-beta peptide levels in various cell types, including those derived from patients [63]. Mepacrine (quinacrine) has emerged as a potential candidate for AD treatment. Although primarily known as an antimalarial drug, recent studies have revealed its ability to reduce amyloid-β production in vitro [64] and Mepacrine (quinacrine) has been found to directly dissociate amyloid plaques in the brains of 5XFAD transgenic mouse models of AD [65].

Gedunin inhibits microglial activation induced by Aβ1–42 oligomers by modulating Nrf2 and NF-κB [66]. Amlodipine, a calcium channel blocker, has shown promising results in phase III clinical trials when combined with losartan and atorvastatin [67]. Researchers have suggested that disulfiram may be repurposed as a therapy for AD by enhancing ADAM10, which acts as an alpha-secretase in neurons [68]. Additionally, Trichostatin A and CGP-60474 showed anti-AD potential in both in silico and in vivo experiments [69,70]. Therefore, the transition to novel candidates, such as DHEC and mepacrine, represents a paradigm shift towards more targeted and potentially more efficacious treatments for AD. These compounds offer alternative mechanisms of action and enhanced safety profiles compared with conventional cholinesterase inhibitors. Hence, it is imperative to subject the pharmaceuticals and organic substances identified in this study to appropriate in vitro and in vivo model testing to comprehensively evaluate their efficacy and safety before proceeding to preclinical evaluation [71,72]. However, a limitation of this study is the use of a single RNA-seq dataset. The decision to exclude other datasets related to AD was made to mitigate potential bias in obtaining precise results, which could arise from variations in patient cohorts, sample numbers, sample types, read depths, and instrumentation in RNA-seq experiments. Nevertheless, our future research plans include collecting data from similar experiments when available in the field of AD research to conduct comprehensive NGKD analyses.

## 5 Conclusions and future directions

In this study, we identified potential pathways, gene signatures, and therapeutic options for elderly patients with dementia, using RNA-seq data and advanced knowledge discovery techniques. However, introducing novel therapies into the complex networks of the brain poses significant challenges, owing to the delicate balance within these networks. To ensure safety and efficacy, it is crucial to understand the potential interactions between various therapies and their implications for brain function. Furthermore, ethical considerations are of utmost importance when dealing with therapies that directly affect the brain and its function. Ongoing advancements in AD research have led to discoveries and breakthroughs, and lifestyle modifications and environmental factors have played crucial roles in disease prevention and management. Innovative therapies extend beyond traditional medications and include noninvasive brain stimulation techniques, gene editing technologies, and dietary interventions, making the holistic management of AD essential. However, the long-term effects of certain therapies on slowing cognitive decline and preventing plaque accumulation remain unclear. Therefore, personalized medicine for AD, tailored to individual genetic and biological profiles, is necessary to effectively treat this debilitating disease.

## CRediT authorship contribution statement

**Hind A. Alkhatabi:** Writing – review & editing, Writing – original draft, Visualization, Validation, Methodology, Investigation, Funding acquisition, Formal analysis, Data curation, Conceptualization. **Peter Natesan Pushparaj:** Writing – review & editing, Writing – original draft, Visualization, Validation, Supervision, Software, Resources, Project administration, Methodology, Investigation, Formal analysis, Data curation, Conceptualization.

## Data availability statement

The RNAseq dataset used in this study (accession number GSE104704) is openly available in the Gene Expression Omnibus (GEO) for download and reuse.

## Declaration of competing interest

The authors declare that they have no known competing financial interests or personal relationships that could have appeared to influence the work reported in this paper.

## Figures and Tables

**figs1:**
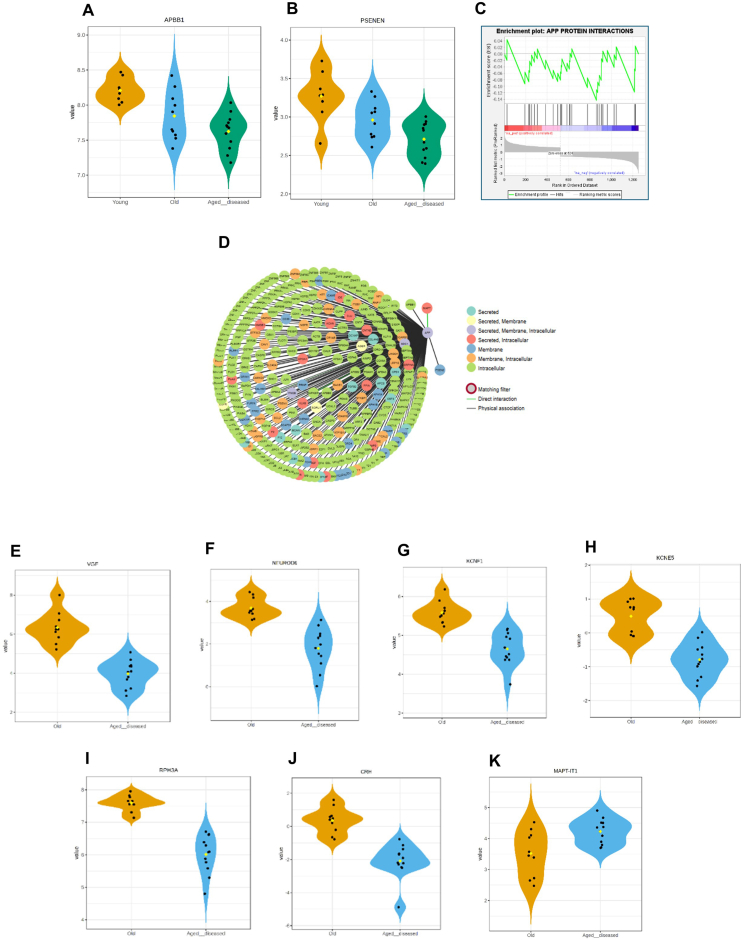


**figs2:**